# Prospective predictors of electronic nicotine delivery system initiation in tobacco naive young adults: A machine learning approach

**DOI:** 10.1016/j.pmedr.2023.102148

**Published:** 2023-02-13

**Authors:** Nkiruka C. Atuegwu, Eric M. Mortensen, Suchitra Krishnan-Sarin, Reinhard C. Laubenbacher, Mark D. Litt

**Affiliations:** a Department of Medicine, University of Connecticut School of Medicine, Farmington, CT 06030, USA; b Department of Psychiatry, Yale University School of Medicine, Connecticut Mental Health Center, 34 Park Street, New Haven, CT 06519, USA; c Laboratory for Systems Medicine, Department of Medicine, University of Florida, Gainesville, FL 32610, USA; d Division of Behavioral Sciences and Community Health, University of Connecticut Health Center, Farmington, CT 06030, USA

**Keywords:** E-cigarette, Electronic nicotine delivery systems, ENDS, Machine learning, Tobacco naïve, Never tobacco users, Vaping, Young adults, Prospective predictors, PATH, Population Assessment of Tobacco and Health survey

## Abstract

The use of electronic nicotine delivery systems (ENDS) is increasing among young adults. However, there are few studies regarding predictors of ENDS initiation in tobacco-naive young adults. Identifying the risk and protective factors of ENDS initiation that are specific to tobacco-naive young adults will enable the creation of targeted policies and prevention programs. This study used machine learning (ML) to create predictive models, identify risk and protective factors for ENDS initiation for tobacco-naive young adults, and the relationship between these predictors and the prediction of ENDS initiation. We used nationally representative data of tobacco-naive young adults in the U.S drawn from the Population Assessment of Tobacco and Health (PATH) longitudinal cohort survey. Respondents were young adults (18–24 years) who had never used any tobacco products in Wave 4 and who completed Waves 4 and 5 interviews. ML techniques were used to create models and determine predictors at 1-year follow-up from Wave 4 data. Among the 2,746 tobacco-naive young adults at baseline, 309 initiated ENDS use at 1-year follow-up. The top five prospective predictors of ENDS initiation were susceptibility to ENDS, increased days of physical exercise specifically designed to strengthen muscles, frequency of social media use, marijuana use and susceptibility to cigarettes. This study identified previously unreported and emerging predictors of ENDS initiation that warrant further investigation and provided comprehensive information on the predictors of ENDS initiation. Furthermore, this study showed that ML is a promising technique that can aid ENDS monitoring and prevention programs.

## 1 Introduction

Electronic nicotine delivery systems (ENDS), commonly known as e-cigarettes, are extremely popular among young adults. Between 2018 and 2019, ENDS use increased from 7.6 % to 9.3 % among 18–24-year-olds (QuickStats, 2019, Cornelius et al., 2020). In 2019, ENDS use was highest among young adults aged 18–24 years compared with other adult age groups, and 56.0 % of these young adults were never cigarette smokers (Cornelius et al., 2020). This use of ENDS by these young adults has major implications for their health because of the nicotine and other toxicants that they contain. ENDS use can lead to nicotine addiction, substance use problems and future addictions (Overbeek et al., 2020).

Some cross sectional studies have identified age, male sex, race, cigarette and alcohol use, history of depressive disorder, sensation seeking, higher perceived benefits of ENDS and low adulthood socioeconomic-status, as being associated with ENDS use (Atuegwu et al., 2020, Kenne et al., 2016, Patel et al., 2016, Roys et al., 2020, Teah and Conner, 2021). There are a few studies on the prospective predictors of ENDS initiation in young adults. These studies found that ENDS use by peers, use of alcohol, marijuana, and tobacco products, exposure to ENDS marketing, perceptions of ENDS being less harmful and addicting than cigarettes, and that ENDS use could aid in smoking cessation were predictors of ENDS initiation (Agarwal et al., 2017, Choi and Forster, 2014, Cooper et al., 2022, Cooper et al., 2018, Loukas et al., 2019, Spindle et al., 2017). These studies examined a few predictors at a time in young adults with different tobacco product use status. However, reasons for ENDS use have been shown to be different for smokers and never cigarette smokers (Biener et al., 2015). Therefore, predictors of ENDS initiation for tobacco-naive young adults may be different from those of young adults who have used other tobacco products. Identifying the risk and protective factors of ENDS initiation that are specific to tobacco-naive young adults will enable the creation of targeted policies and prevention programs.

This study is among the first to use machine learning (ML) models to do a systematic study of the associations between a large number of variables and ENDS initiation in a nationally representative sample. This study adds to the existing literature on ENDS initiation by using ML techniques to provide comprehensive information on the prospective predictors and groups of tobacco-naive young adults who are most likely to try, or initiate ENDS use in the future.

Our ML analysis uses tree-based algorithms which are effective for large observational data. The ML techniques employed can accommodate multiple predictors and complex relationships, and can identify covariate patterns that predict outcomes better than prespecified models. These ML techniques also reduce errors due to statistical model misspecification and can capture non-linear relationships and interactions between predictors and the endpoint, ENDS initiation (Bzdok et al., 2018, Mooney et al., 2021, Qiu et al., 2022). Therefore, our ML analysis can identify the important predictors that may be focused on for targeted polices and prevention programs. ML has been used in ENDS research (Choi et al., 2021, Fu et al., 2021, Han et al., 2021), including in young adults (Atuegwu et al., 2020). The goal in this study was to use ML to create predictive models and identify multiple prospective predictors of ENDS initiation in tobacco-naive young adults.

## 2 Method

### 2.1 Data and study sample

Predictors of ENDS use identified for earlier generations of ENDS users may be different from those identified today due to changing trends in attitudes, newer products, and policy interventions. Therefore, to identify current prospective predictors of ENDS initiation in tobacco-naive young adults, we used the recent, publicly available Waves of the Population Assessment of Tobacco and Health (PATH) data: Wave 4 (12/2016–1/2018) and Wave 5 (12/2018–11/2019) (United States Department of Health Human Services et al., 2021).

The adult PATH survey is a nationally representative, longitudinal cohort study of the tobacco use patterns and health of civilian non-institutionalized adults in the US. Surveys for new Waves are conducted approximately a year after the prior Wave. Data from young adults aged 18–24 years in Wave 4 who reported never using any tobacco products, and who completed Waves 4 and 5 interviews were used for the analysis. Never tobacco users reported never use of the following tobacco products: cigarettes, ENDS, cigars, cigarillos, pipe, hookah, snus, smokeless and dissolvable tobacco. ENDS include e-cigarettes, e-cigars, e-pipes and e-hookah. This study used the publicly available data of the PATH project and was deemed exempt by the Institutional Review Board.

Young adults who have never used any tobacco product at Wave 4 and who reported any ENDS use at Wave 5 were classified as ENDS initiators. The comparison group for the outcome were those young adults who reported never using ENDS in both Waves. There were 2,746 respondents available for the analysis. The population weighted proportions of females, Whites, Hispanics and ENDS initiators were 53.8 %, 67.8 %, 21.3 % and 9.0 % respectively. Respondents’ baseline descriptive statistics of sociodemographic and substance use are shown in Table 1.

**Table 1:** Descriptive statistics of the baseline (Wave 4) sociodemographic and substance use stratified by ENDS initiation at Wave 5^a^.

	All respondents (N = 2746)	Not ENDS initiator (N = 2437)	ENDS initiators (N = 309)
Gender			
Male	1238 (46.2)	1080(45.6)	158(52.0)
Female	1508 (53.8)	1357 (54.4)	151 (48.0)
Race			
White alone	1813 (67.8)	1594 (67.4)	219 (72.5)
Black alone	525 (16.1)	479 (16.4)	46 (12.7)
Other^b^	408 (16.1)	364 (16.2)	44 (14.8)
Hispanic			
Yes	790 (21.3)	695 (21.1)	95 (23.2)
No	1956 (78.7)	1742 (78.9)	214 (76.8)
Currently enrolled in a degree program			
Yes	1344(50.3)	1173 (49.4)	171 (59.2)
No	1393(49.5)	1257 (50.4)	136 (40.2)
Anyone who lives with you now who uses tobacco			
Cigarettes, cigars, cigarillos or filtered cigars and pipe tobacco	461 (17.2)	391 (16.7)	70 (22.2)
E-products exclusively	42 (1.6)	35 (1.5)	7 (2.1)^c^
Other tobacco products, including smokeless, snus and hookah	78 (2.5)	63 (2.3)	15 (4.2)
No one living in the home uses tobacco	2040 (73.7)	1833 (74.2)	207 (67.9)
Currently live with a spouse or romantic partner			
Yes	245 (13.3)	237 (14.2)	8 (3.6)^c^
No	1999 (71.1)	1767 (70.5)	232 (77.1)
Used marijuana in the past 12 months			
Yes	227 (7.6)	166 (6.5)	61 (19.8)
No	2518 (92.3)	2270 (93.5)	248 (80.2)
Used alcohol in the past 12 months			
Yes	1178 (47.9)	1001 (46.6)	177 (61.6)
No	1565 (51.9)	1433 (53.3)	132 (38.4)

a Data is expressed as unweighted number (weighted percentage) of respondents.

b Other race includes respondents who are American Indian or Alaska Native, Asian Indian, Chinese, Filipino, Japanese, Korean, Vietnamese, Other Asian, Native Hawaiian, Guamanian or Chamorro, Samoan, Other Pacific Islander and multiracial.

c Relative standard error (RSE) > 30 %, therefore weighted percentages may not be reliable.

### 2.2 Independent variables

The PATH dataset contains > 2000 variables, including demographics, tobacco and substance use, advertising exposures, risk perceptions, and peer and family influences. Details about PATH variables are available elsewhere (Hyland et al., 2017, United States Department of Health Human Services, 2021).

### 2.3 Model development and statistical analysis

After preprocessing the data (Supplementary Methods), 242 variables were available for ML analysis. The Extreme gradient boosting (XGBoost) algorithm (Chen and Guestrin, 2016) was used to create the prediction models and the SHapley Additive exPlanations (SHAP) (Lundberg and Lee, 2017) algorithm was used to interpret the final models (Supplementary Methods). To corroborate the findings from ML, multivariable logistic regression models for the outcome and the predictors identified using ML were created. These models adjusted for gender, race and ethnicity and accounted for the PATH complex design.

## 3 Results

Model performance was calculated with the Area under the Receiver Operating Characteristic curve (AUC) and the Area under the Precision Recall Curve (AUCPR). The maximum AUC and AUCPR for the XGBoost classifier were 0.82 (mean of 0.73; standard deviation of 0.05) and 0.48 (mean of 0.30; standard deviation of 0.06) respectively. The baseline AUCPR and AUC for the analysis were 0.11 (unweighted prevalence of ENDS initiators) and 0.5 respectively. AUC and AUPRC above the baseline values indicate that the model can discriminate between ENDS initiators and non-initiators (Saito and Rehmsmeier, 2015). Descriptive statistics of influential baseline variables on the prediction of ENDS initiation selected by ML are in Supplementary Table 1.

**Fig. 1:**
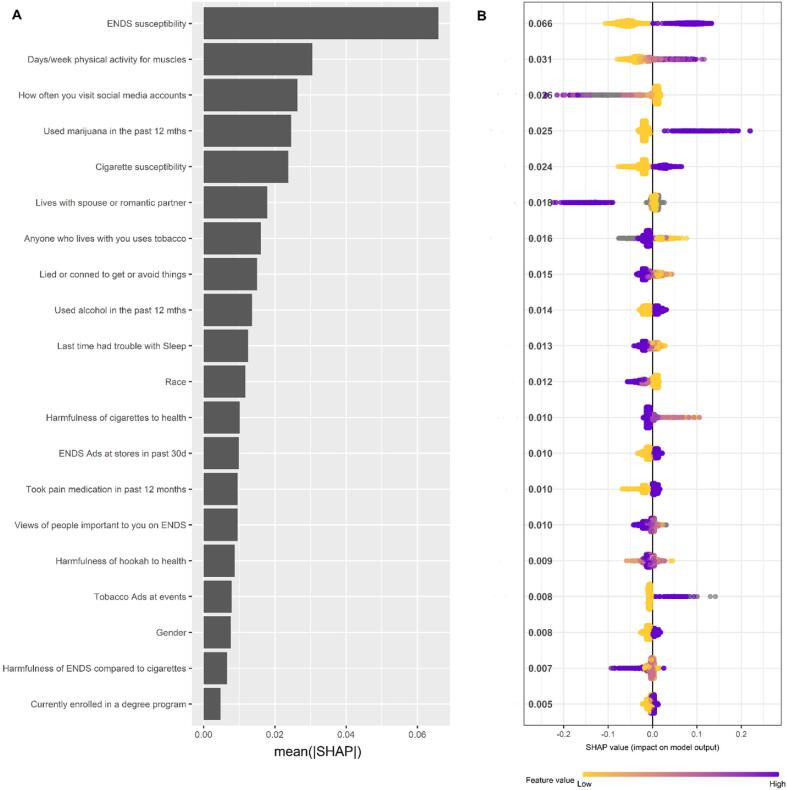
SHAP global importance (A) and summary plot (B) of the prospective predictors of ENDS initiation at 1-year follow up for the top 20 variables. Global importance ranks predictors in order of their importance to the prediction of ENDS initiation. The variable importance is determined by the mean of the absolute value of the SHAP values shown in the x-axis in (A) and y-axis in (B). The higher the mean of the absolute SHAP value, the more important the variable is to the prediction of ENDS initiation. In (B) each dot is a respondent, the color of the dot represents the value of the predictor and the horizontal location of the dot shows the SHAP value. A negative SHAP value indicates that the predictor contributed to the respondent not initiating ENDS while a positive SHAP value indicates that the predictor contributed to the respondent initiating ENDS.

**Fig. 2:**
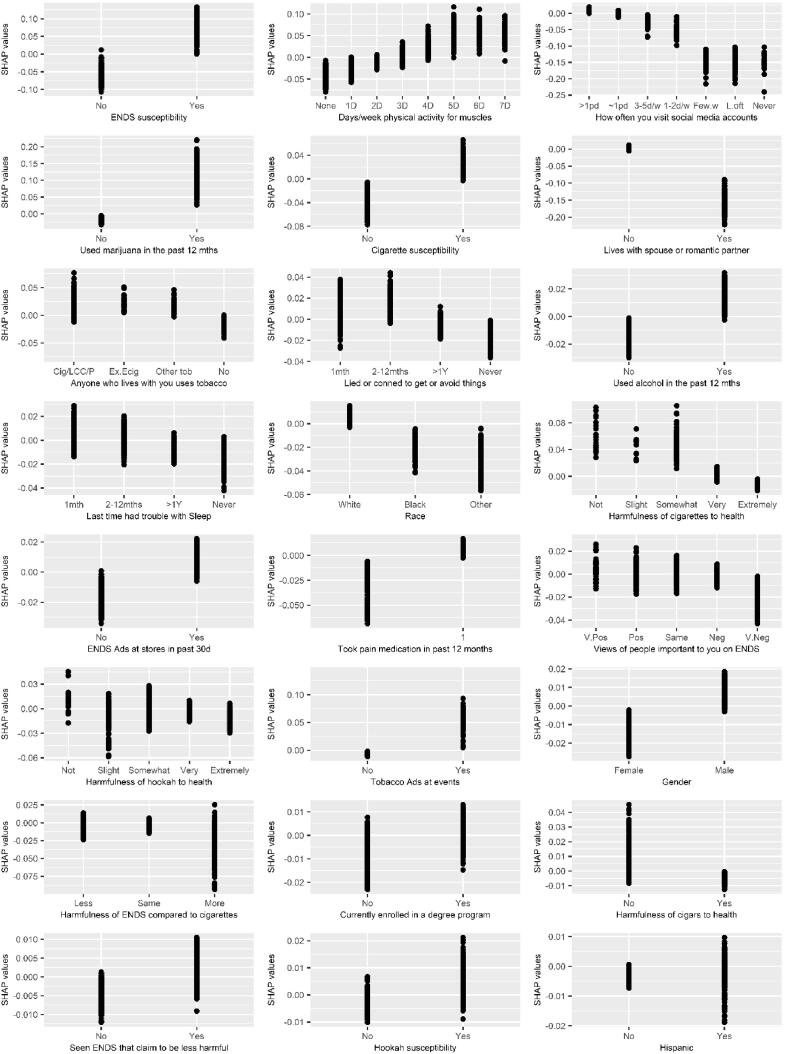
SHAP dependence plots for the predictors of ENDS initiation. The x and y-axes are the value and SHAP values of the predictors respectively. Positive SHAP values indicate increased risk of ENDS initiation and negative SHAP values indicate decreased risk of ENDS initiation.

The top 20 predictors based on SHAP values are shown in Fig. 1A (Global importance) and 1B (Summary plot). SHAP dependence plots (Fig. 2) show the relationship (e.g., complex) between the variable and the prediction of the outcome. Positive SHAP values indicate increased risk of initiating ENDS while negative SHAP values indicate decreased risk of initiating ENDS.

The most influential variable was susceptibility to ENDS. As described elsewhere (Nicksic and Barnes, 2019, Pérez et al., 2021, Pierce et al., 2018, Pierce et al., 2017), susceptibility to ENDS was determined by reporting “Definitely yes”,“Probably yes” or “Probably not” to the following questions: “Think you will use an electronic nicotine product in the next year”, “Think you will try an electronic nicotine product soon”, “Would try an electronic nicotine product if one of your best friends offered it to you” and “A little curious” “Somewhat curious” or “Very curious” to the question “Ever been curious about using an electronic nicotine product”. Non-susceptible young adults answered “Definitely not” to the first three questions and “Not at all curious” to the last question. Young adults who recorded any other response combinations were considered susceptible. Susceptibility to cigarettes and hookah were determined with similar questions. Reporting ENDS susceptibility was associated with positive SHAP values and therefore increased risk of initiating ENDS, while reporting no ENDS susceptibility was associated with negative SHAP values and therefore decreased risk of initiating ENDS (Fig. 1A and 2). We grouped the influential variables identified with SHAP (Fig. 2) into categories for clarity.

### 3.1 Substance use and susceptibility to tobacco

Susceptibility to ENDS, cigarettes and hookah, and using marijuana and alcohol in the past 12 months was associated with positive SHAP values. Also, reporting taking pain medication in the past 12 months was associated with positive SHAP values.

### 3.2 Physical activity

The SHAP values for physical exercise specifically designed to strengthen muscles (not including cardio) were negative for no days of strength training and increased with increasing days of strength training with positive values occurring at ≥ 4 days a week.

### 3.3 Internalizing and externalizing problems

Responding “never” to: “lied or conned to get things or avoid doing something” (externalizing problem) and “sleep trouble” (internalizing problems) (Conway et al., 2018), were associated with negative SHAP values.

### 3.4 Media use and exposure to advertisement

The SHAP values for frequency of use of social media were positive for reporting use of social media more than once a day and decreased with decreasing frequency of social media use. Noticing ENDS being advertised at gas stations and stores, noticing tobacco products being advertised at events, and seeing any advertisements claiming that ENDS were less harmful than cigarettes was associated with positive SHAP values.

### 3.5 Demographics, social influence, and beliefs about tobacco products

Living with a spouse/romantic partner, reporting being of Black or Other race, female gender, believing that cigarettes, hookah and cigars were harmful to health, not living with a tobacco user, and having important people with very negative views on ENDS were associated with negative SHAP values. In contrast, reporting current enrollment in a degree program was associated with positive SHAP values.

### 3.6 Multivariable logistic regression models

The odds ratios for most of the predictors identified with ML showed statistically significant associations with ENDS initiation (Supplementary Table 2). We however found no statistically significant association between race, gender and ENDS initiation. A possible explanation may be that the relationship between gender, race and ENDS initiation is more complex than our prespecified logistic regression models. However, our ML approach may have been able to capture this relationship because of its ability to consider non-linear relationships between the predictors and prediction of the outcome.

### 3.7 Variable interactions

The magnitude of the SHAP values for the pairwise interactions between variables in the model are shown in Supplementary Fig. 1. The most notable interaction occurred between “susceptibility to ENDS” and “susceptible to cigarettes”. Respondents with both of these positive risk factors had even greater predicted risk of initiating ENDS (Supplementary Fig. 2A and C). We also identified some other pairwise variable interactions. The interaction between use of marijuana in the past 12 months and reporting susceptibility to ENDS showed an increased predicted risk of ENDS initiation for young adults who reported no susceptibility to ENDS but had used marijuana (Supplementary Fig. 2D and 2F). The interaction between physical activity designed to strengthen muscles and ENDS susceptibility showed a greater predicted risk of ENDS initiation for young adults who were susceptible to ENDS and who also frequently engaged in physical exercise to strengthen muscles (Supplementary Fig. 2G and 2I).

## 4 Discussion

ML techniques were used to create predictive models and identify multiple risk and protective factors of ENDS initiation from a large number of baseline variables at 1-year follow-up in a nationally representative sample of tobacco-naive young adults. Several predictors of ENDS initiation were identified, and these were ranked in order of their importance to the prediction. The predictors identified with ML were also corroborated with multivariable logistic regression models.

Some of the predictors identified with ML aligned with those previously reported in the literature. Similar to our results from the ML analysis, other investigators also noted that respondents who reported race as Black or Other, and gender as female were less likely to initiate ENDS (Kenne et al., 2016). Also, ever use of alcohol and marijuana, living with a tobacco user, and increased exposure to ENDS and other tobacco product marketing have been shown to be predictors of ENDS initiation in adults (Cooper et al., 2022, Nicksic et al., 2017, Villanti et al., 2015).

The most influential predictor of ENDS initiation was reporting any susceptibility to ENDS. While susceptibility to ENDS has been shown to be associated with ENDS initiation in youth (Bold et al., 2020), to the best of our knowledge this has not been reported in tobacco-naive young adults. Susceptibility to cigarettes was also identified as a predictor of ENDS initiation. This suggests that tobacco-naive young adults, who are curious about smoking or have the intent to smoke, are also more likely to initiate ENDS. This could have implications for policies that have strict bans on ENDS while leaving cigarettes accessible to these young adults. Perhaps, some of these young adults may move on to cigarettes if no ENDS are available. Conversely, ENDS may also lead to future use of cigarettes (Bold et al., 2018). Furthermore, our analysis found that tobacco-naive young adults who were susceptible to both ENDS and cigarettes had further increased risk of initiating ENDS. Intervention strategies to reduce ENDS initiation should identify and target young adults who are susceptible to ENDS as well as other tobacco products. Advertisements in media, and schools, might preempt nicotine use by appealing directly to those who are curious but not using nicotine products.

We found that young adults who used marijuana in the past 12 months were more likely to initiate ENDS. We also found that marijuana use in the past 12 months was associated with increased risk of ENDS initiation among young adults who reported no susceptibility to ENDS. These preliminary findings indicate that while reporting no susceptibility to ENDS was protective against initiating ENDS, the use of marijuana by these young adults may have increased the risk of ENDS initiation for these young adults. Additional studies are needed to understand the association between marijuana use and ENDS initiation especially for young adults who report no susceptibility to ENDS.

We found that young adults who used social media accounts more than once a day had increased risk of initiating ENDS, and that this risk decreases as the frequency of use of social media decreases. A possible explanation may be that the use of social media exposes young adults to ENDS and tobacco advertisement as well as other tobacco related content from peers, all of which could increase susceptibility to ENDS (Yang et al., 2021). Efforts to regulate ENDS and tobacco related messages on social media, and the placement of anti-ENDS messages on social media sites may reduce ENDS initiation.

The current study extends the literature on ENDS initiation by identifying ≥ 4 days a week of physical activity designed to strengthen muscles as a predictor of ENDS initiation. We also found further increased risk of ENDS initiation for young adults who were susceptible to ENDS use and who frequently engaged in exercise to strengthen muscles. It is plausible that young adults who are trying to gain muscle mass and improve their physique are using nicotine supplied via ENDS to suppress appetite and reduce body fat. Nicotine has been shown to reduce body fat and decrease food intake (Calarco and Picciotto, 2020). Other explanations could be that young adults who are drawn to the ‘novelty’ of muscle building may also be drawn to the ‘novelty’ of ENDS use. Also, these young adults may be subject to more pressure in gyms to use ENDS. Further studies are required to understand the association between frequency of muscle building exercises and ENDS initiation including the interaction with ENDS susceptibility, and to validate if this may be an emerging group of young adults who are vulnerable to ENDS use.

Additionally, we found that young adults who used pain medication in the past 12 months were more likely to initiate ENDS. Perhaps these young adults may be using ENDS for some pain relief due to the analgesic effects of nicotine (Ditre et al., 2016). It is important to understand the association between pain and ENDS initiation especially in tobacco-naive young adults. We also found that young adults who were currently enrolled in a degree program were more likely to initiate ENDS. This is in contrast with a study that showed that non-college young adults had higher prevalence of ENDS use (Buu et al., 2020a, Buu et al., 2020b). The difference may be due to the data used. The earlier study used Wave 1 of PATH (2013–2014). The current study, using a later PATH Wave, may be capturing a more recent trend in ENDS use among college students. A recent report by the American College Health Association showed that ENDS are the most commonly used tobacco product among young adults transitioning to and on college campuses (American College Health Association, 2021). Similarly, the Monitoring The Future (MTF) study showed a 6 % to 19 % increase from 2017 to 2020 in past 30-day nicotine vaping for college students (Schulenberg et al., 2021), and a higher annual prevalence of nicotine vaping among college than non-college students from 2018 to 2020. Strategies such as targeted campaigns, promoting awareness of ENDS use with student health providers, and promoting tobacco-free campus policies may reduce ENDS initiation in colleges (American College Health Association, 2021, Escoto et al., 2021).

Our analysis showed that young adults who did not report internalizing and externalizing problems were less likely to initiate ENDS. Internalizing and externalizing problems are indicative of mental health problems (Riehm et al., 2019). This contrasts with a study that showed no association between reporting internalizing or externalizing problems and ENDS initiation in young adults (Cooper et al., 2022). This difference may be due to the sample of young adults used. Our analysis focused on tobacco-naive young adults. Other studies however have shown that reporting higher numbers of internalizing and externalizing problems was associated with higher risks of initiating ENDS in youth (Buu et al., 2020a, Buu et al., 2020b, Cooper et al., 2022). We also found that young adults who lived with spouses/romantic partners were less likely to initiate ENDS. This may be because these young adults were less likely to engage in risky behaviors such as ENDS use. Intimate relationships have been shown to have a significant effect on health behaviors (Umberson et al., 2010). Our analysis found that young adults who had negative views on tobacco, did not live with tobacco users, and had a social network of people who were not supportive of ENDS use, were less likely to initiate ENDS. Youth and young adults who had friends who smoked cigarettes or had used ENDS were more likely to initiate ENDS themselves (Jayakumar et al., 2020). ENDS prevention programs should identify young adults with internalizing and externalizing problems for targeted interventions, should emphasize the negative effects of tobacco products, and should encourage establishing a social network of non-tobacco users.

A strength of the current study was the use of respondents who were nationally representative of tobacco-naiïve young adults in the U.S. Additionally, ML removed the biases in preselected models of ENDS initiation. We did not prespecify the predictors and models of ENDS initiation, rather we used ML to simultaneously consider many variables, identify predictors including those previously unknown, and identify nonlinear relationships and interactions between the predictors and the prediction of ENDS initiation. This decreased the possibility of statistical misclassification, and the omission of predictors. We also used multivariable logistic regression models to independently corroborate the findings from ML. Furthermore, our findings showed the predictors that may have the most impact on ENDS initiation in tobacco-naive young adults and may provide possible areas that public health officials could concentrate on to decrease ENDS initiation in tobacco-naive young adults.

This study has some limitations. The data are based on self-report and may have recall bias. We cannot establish causality from our analysis. We could not include some potential predictors such as the age of the respondents, “sensation seeking” and a diagnosis of depression in the model because they were not available in the public adult PATH survey. There may be other confounders that can affect the identified predictors. While we feel that the sample size of young adults is sufficient to carry out the ML analysis, a larger sample size may increase the accuracy of the models. We created the ML models on unweighted PATH data, because the ML algorithms could not account for the PATH complex design. However, our findings are still relevant and were corroborated with logistic regression models that incorporated the PATH complex design and weights. There is also a possibility of spurious findings. We reduced the possibility of spuriousness because the ML algorithms considered all the variables and their interactions simultaneously and not serially. Also, the use of repeated CV and different ML algorithms reduced the dependence of the results on one ML or iteration of a ML algorithm, which reduced the likelihood of spurious findings. Furthermore, our findings were corroborated with logistic regression models. Respondents who initiate ENDS use may become experimental or frequent users. Future work should create ML models for these types of ENDS use.

In conclusion, we showed that ML can identify predictors and the complex relationships between these predictors and ENDS initiation. This indicates that ML may be a promising technique for ENDS monitoring and prevention programs, and may identify emerging predictors of ENDS use. Our findings if validated may have implications for policies and programs designed to prevent ENDS initiation in young adults.

## Funding

Dr. Atuegwu receives support from Robert E. Leet and Clara Guthrie Patterson Trust Mentored Research grant.

## CRediT authorship contribution statement

**Nkiruka C. Atuegwu:** Conceptualization, Methodology, Formal analysis, Writing – original draft, Writing – review & editing, Visualization. **Eric M. Mortensen:** Writing – review & editing. **Suchitra Krishnan-Sarin:** Writing – review & editing. **Reinhard C. Laubenbacher:** Writing – review & editing. **Mark D. Litt:** Writing – review & editing.

## Declaration of Competing Interest

The authors declare that they have no known competing financial interests or personal relationships that could have appeared to influence the work reported in this paper.
