# Supplementary material for: Prospective predictors of electronic nicotine delivery system initiation in tobacco naive young adults: A machine learning approach
Source: Prev Med Rep. 2023 Feb 13;32:102148. doi: 10.1016/j.pmedr.2023.102148 (PMC9971268; doi:10.1016/j.pmedr.2023.102148)
Supplement: Supplementary data 1 [file mmc1.docx]

*
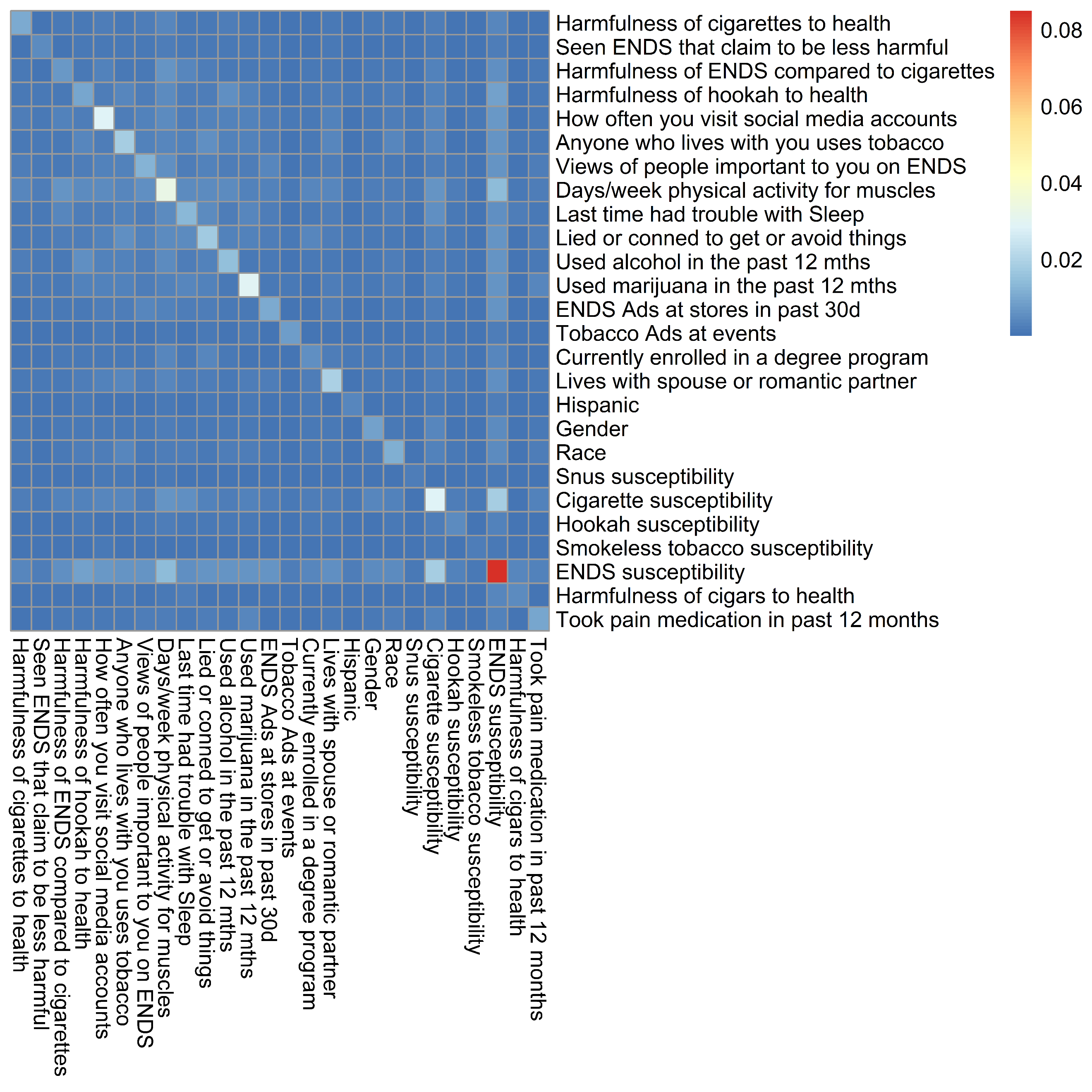
*

**Supplementary Figure 1:** Heat map showing the sum of the absolute value of the SHAP pairwise interactions values for the variables for all the respondents.
