# Supplementary material for: Prospective predictors of electronic nicotine delivery system initiation in tobacco naive young adults: A machine learning approach
Source: Prev Med Rep. 2023 Feb 13;32:102148. doi: 10.1016/j.pmedr.2023.102148 (PMC9971268; doi:10.1016/j.pmedr.2023.102148)
Supplement: Supplementary data 2 [file mmc2.docx]

***
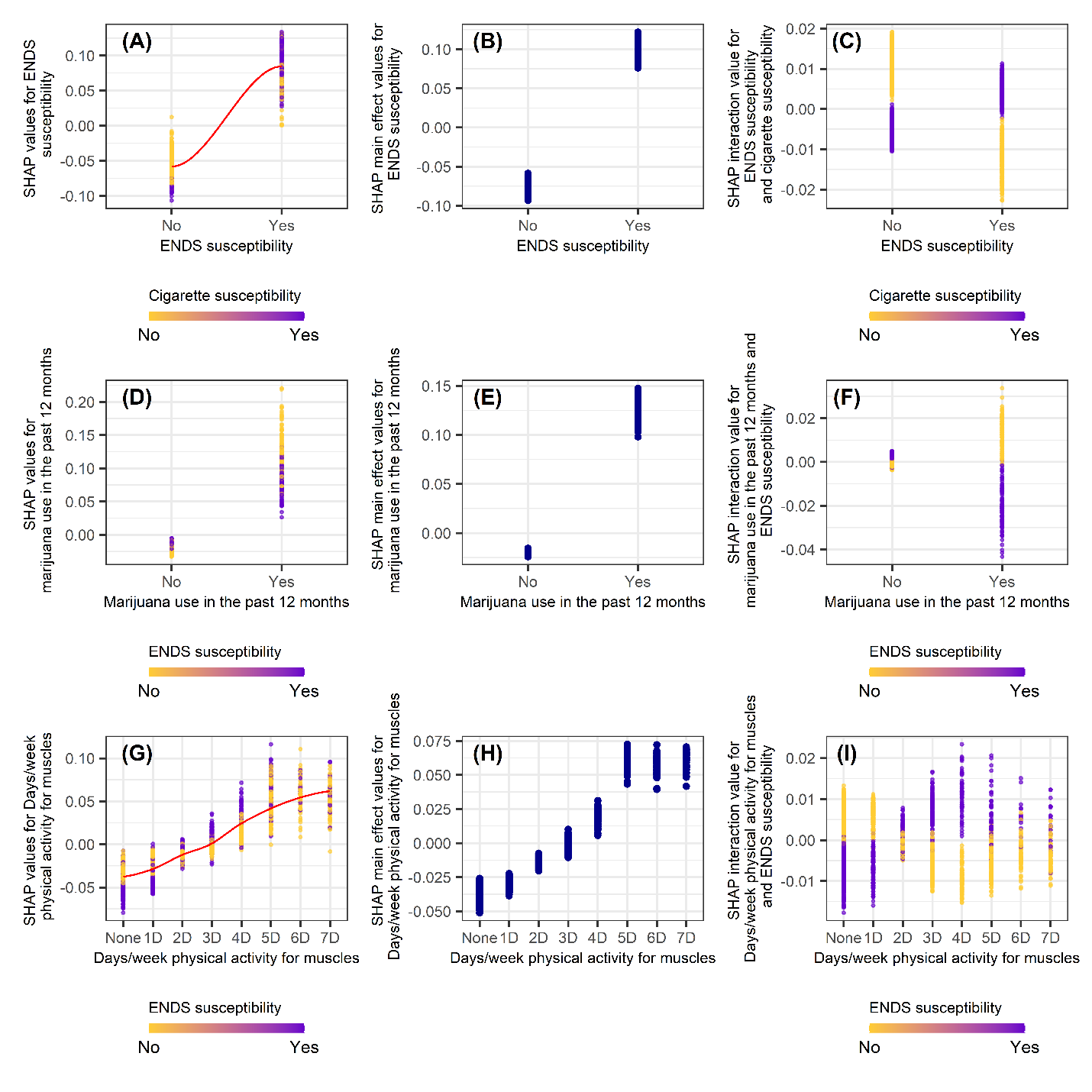
***

**Supplementary Figure 2:** Panel A shows how the SHAP values of ENDS susceptibility depend on cigarette susceptibility. Panel B, E and H shows the main effect (no interactions) of ENDS susceptibility, marijuana use in the past 12 months and days/week of physical activity for muscles respectively. Panel C show the SHAP interaction value for ENDS and cigarette susceptibility. Panel D shows how the SHAP values of marijuana use in the past 12 months depend on ENDS susceptibility. Panel F show the interaction values for marijuana use in the past 12 months and ENDS susceptibility. Panel G show how the SHAP values of days/week of physical exercise vary with ENDS susceptibility. Panel I show interaction value of days/week of physical exercise and ENDS susceptibility. Positive SHAP values indicate increased risk and negative SHAP values indicate decreased risk of ENDS initiation.
