# Supplementary material for: Prospective predictors of electronic nicotine delivery system initiation in tobacco naive young adults: A machine learning approach
Source: Prev Med Rep. 2023 Feb 13;32:102148. doi: 10.1016/j.pmedr.2023.102148 (PMC9971268; doi:10.1016/j.pmedr.2023.102148)
Supplement: Supplementary data 3 [file mmc3.docx]

**Supplementary Methods**

**Model development and statistical analysis**

Variables with no research implications (e.g., order of randomization), variables with low variances (similar in ≥ 99% of respondents), and variables that did not pertain to ≥ 70% of the study sample were removed. This was done to remove the variables that were not relevant to the present study sample. Missing data in PATH are mainly due to skip logic. Missing data that could be determined from previous answers by the respondents’ were replaced with the appropriate response. Missing data due to a question not being applicable to the respondent were differentiated from missing data due to non-response and replaced with a categorical value. Missing data due to non-response included data that was missing due to “data removed per respondent request, improbable answer, don't know or refused”. These were imputed with mode imputation (Tsikriktsis, 2005). After preprocessing the data, 242 variables were available for initial variable selection.

The percentage of missing data for the variables ranged from 0 to 26% (median of 0.25%). Only 18 of the 242 variables had missing data greater than 5%. The dataset with imputed missing variables was used only for initial variable selection because the variable selection algorithm required a dataset without any missing data. Imputed data was not used to build the final models because the algorithm used to create the final models (Extreme gradient boosting (XGBoost) ) estimates missing data using a sparsity-aware split finding algorithm, which learns the best direction to classify missing data in the decision tree (Chen and Guestrin, 2016). The performance of an XGBoost classifier trained on datasets with missing data is statistically the same or superior to an XGBoost classifier trained on imputed datasets (Ergul Aydin and Kamisli Ozturk, 2021; Rusdah and Murfi, 2020; Vaid et al., 2021). However, we compared the performance of XGBoost on dataset with and without the imputed data and the accuracy was slightly better for the non-imputed data. Therefore, we used the non-imputed dataset for our analysis.

The Boruta algorithm was used for initial variable selection (Kursa and Rudnicki, 2010). It was used to select variables that were associated with the outcome, thereby removing non-informative variables that could degrade the accuracy of the models (Guyon and Elisseeff, 2003). Race, gender and ethnicity, which are non-modifiable demographic variables (Stallings-Smith and Ballantyne, 2019), were always included as variables in the prediction models. The PATH dataset categorized age into groups with 18-24 years as an age group; therefore, we could not include age as a variable in the models.

To estimate the prediction error of the ML model, we used a nested cross validation (CV) scheme with an outer repeated stratified 10-fold CV and an inner stratified 10-fold CV for hyperparameter optimization. CV reduces the bias in the estimation of the prediction error and increases the confidence that the results obtained can be replicated beyond the dataset used for the study (Koul et al., 2018; Rodriguez et al., 2010). Initial variable selection, hyperparameter optimization and the prediction models were performed/created inside every training set of the outer 10-fold CV in order to produce unbiased estimates of the prediction error (Varma and Simon, 2006). The 10-fold CV was repeated 25 times and the performance of the classifier in each CV was calculated via the Area under the Receiver Operating Characteristic curve (AUC) and Area under the Precision Recall Curve (AUCPR) (Saito and Rehmsmeier, 2015).

XGBoost, a modified boosted tree algorithm, was used to create the prediction models with the variables selected during initial variable selection (Chen and Guestrin, 2016). XGBoost can capture non-linear relationships and interaction effects between variables and the outcome by splitting on different features in the same tree (Qiu et al., 2022). XGBoost is not affected by multicollinearity, can handle class imbalance in the data (which can reduce the accuracy of models), and can estimate missing data. Bayesian optimization algorithm was used for hyperparameter optimization for the XGBoost algorithm (Snoek et al., 2012).

Different sets of variables were selected in each CV, therefore, variables selected in ≥ 30% of all the CVs were selected as potential final variables. This was done to select important predictors while reducing the chances of selecting spurious variables. Hierarchical clustering (Chavent et al., 2012) was used to cluster these selected variables and identify highly correlated variables. Variables that clustered together were either combined to create a new variable or replaced with a variable that represented the cluster. The final models were interpreted using SHapley Additive exPlanations (SHAP) (Lundberg and Lee, 2017). The clustering of similar variables was done to increase the accuracy of the interpretation and the ranking of the variables with SHAP.

To avoid the errors and limitations due to a single application of an ML algorithm (Kursa, 2014), we created 250 predictive models with XGBoost on the full dataset. SHAP values were calculated for each predictive model and the mean SHAP values across all the models were calculated. SHAP values are consistent calculations of each variable’s contribution to the model’s prediction. SHAP values show how the variables affect the model’s predictions, including the magnitude and direction of the prediction. Additionally, SHAP values can be decomposed into the main effects (no interactions) and the effect of interactions with other variables in the prediction models and as such can be used to study the pairwise interactions between the variables in the model (Lundberg et al., 2020; Lundberg et al., 2018; Lundberg and Lee, 2017).

To corroborate the findings from ML, we created multivariable logistic regression models for the outcome and the predictors identified using ML. These models adjusted for gender, race and ethnicity and accounted for PATH complex design with the use of weights and variance estimation procedures. R (version 3.6.1) was used for the data analysis.

**REFERENCES**

Chavent, M., Kuentz-Simonet, V., Liquet, B., Saracco, J., 2012. ClustOfVar: An R Package for the Clustering of Variables. Journal of Statistical Software 50:1 - 16.

Chen, T., Guestrin, C., 2016. XGBoost: A Scalable Tree Boosting System, Proceedings of the 22nd ACM SIGKDD International Conference on Knowledge Discovery and Data Mining. ACM, San Francisco, California, USA, pp. 785-94.

Ergul Aydin, Z., Kamisli Ozturk, Z., 2021. Performance Analysis of XGBoost Classifier with Missing Data.

Guyon, I., Elisseeff, A., 2003. An introduction to variable and feature selection. J. Mach. Learn. Res. 3:1157–82.

Koul, A., Becchio, C., Cavallo, A., 2018. Cross-Validation Approaches for Replicability in Psychology. Frontiers in Psychology 9.

Kursa, M.B., 2014. Robustness of Random Forest-based gene selection methods. BMC Bioinformatics 15:8.

Kursa, M.B., Rudnicki, W.R., 2010. Feature Selection with the Boruta Package. Journal of Statistical Software 36:13.

Lundberg, S.M., Erion, G., Chen, H., DeGrave, A., Prutkin, J.M., Nair, B., Katz, R., Himmelfarb, J., Bansal, N., et al., 2020. From local explanations to global understanding with explainable AI for trees. Nature Machine Intelligence 2:56-67.

Lundberg, S.M., Erion, G.G., Lee, S.-I., 2018. Consistent Individualized Feature Attribution for Tree Ensembles. ArXiv abs/1802.03888.

Lundberg, S.M., Lee, S.-I., 2017. A Unified Approach to Interpreting Model Predictions, NIPS.

Qiu, W., Chen, H., Dincer, A.B., Lundberg, S., Kaeberlein, M., Lee, S.-I., 2022. Interpretable machine learning prediction of all-cause mortality. Communications Medicine 2:125.

Rodriguez, J.D., Perez, A., Lozano, J.A., 2010. Sensitivity Analysis of k-Fold Cross Validation in Prediction Error Estimation. IEEE Transactions on Pattern Analysis and Machine Intelligence 32:569-75.

Rusdah, D.A., Murfi, H., 2020. XGBoost in handling missing values for life insurance risk prediction. SN Applied Sciences 2:1336.

Saito, T., Rehmsmeier, M., 2015. The precision-recall plot is more informative than the ROC plot when evaluating binary classifiers on imbalanced datasets. PloS one 10:e0118432.

Snoek, J., Larochelle, H., Adams, R., 2012. Practical Bayesian Optimization of Machine Learning Algorithms. Advances in Neural Information Processing Systems 4.

Stallings-Smith, S., Ballantyne, T., 2019. Ever Use of E-Cigarettes Among Adults in the United States: A Cross-Sectional Study of Sociodemographic Factors. Inquiry : a journal of medical care organization, provision and financing 56:46958019864479.

Tsikriktsis, N., 2005. A Review of Techniques for Treating Missing Data in OM Survey Research. Journal of Operations Management 24:53-62.

Vaid, A., Chan, L., Chaudhary, K., Jaladanki, S.K., Paranjpe, I., Russak, A., Kia, A., Timsina, P., Levin, M.A., et al., 2021. Predictive Approaches for Acute Dialysis Requirement and Death in COVID-19. Clinical Journal of the American Society of Nephrology 16:1158-68.

Varma, S., Simon, R., 2006. Bias in error estimation when using cross-validation for model selection. BMC Bioinformatics 7:91.
