# Supplementary material for: Prospective predictors of electronic nicotine delivery system initiation in tobacco naive young adults: A machine learning approach
Source: Prev Med Rep. 2023 Feb 13;32:102148. doi: 10.1016/j.pmedr.2023.102148 (PMC9971268; doi:10.1016/j.pmedr.2023.102148)
Supplement: Supplementary data 4 [file mmc4.docx]

**Supplementary Table 1:** Descriptive statistics of the baseline (Wave 4) significant predictors selected using ML stratified by ENDS initiation at Wave 5^a^.

|  | Not ENDS initiator  (N = 2437) | ENDS initiator  (N = 309) |
| --- | --- | --- |
| **ENDS susceptibility** |  |  |
| Yes | 637 (26.7) | 167 (52.5) |
| No | 1800 (73.3) | 142 (47.5) |
| **How many days of physical activities specifically designed to strengthen your muscles** |  |  |
| None | 1034 (42.9) | 105 (31.7) |
| 1 day per week | 317 (13.0) | 37 (10.1) |
| 2 days per week | 324 (13.0) | 40 (12.8) |
| 3 days per week | 303 (12.8) | 43 (16.0) |
| 4 days per week | 165 (6.3) | 24 (8.3) |
| 5 days per week | 157 (6.3) | 38 (14.0) |
| 6 days per week | 62 (2.7) | 10 (3.7)^b^ |
| 7 days per week | 68 (2.7) | 12 (3.5)^b^ |
| **How often visit social media accounts** |  |  |
| More than once a day | 1580 (65.3) | 232 (76.3) |
| About once a day | 306 (12.5) | 40 (12.9) |
| 3 - 5 days a week | 144 (5.6) | 13 (3.5) |
| 1 - 2 days a week | 74 (3.0) | 9 (2.6)^b^ |
| Every few weeks | 51 (2.0) | 1 (0.2)^b^ |
| Less often | 62 (2.5) | 2 (0.3)^b^ |
| Never | 217 (8.9) | 12 (4.2)^b^ |
| **Used marijuana in the past 12 months** |  |  |
| Yes | 166 (6.5) | 61 (19.8) |
| No | 2270 (93.5) | 248 (80.2) |
| **Cigarette Susceptibility** |  |  |
| Yes | 591 (23.8) | 144 (45.2) |
| No | 1846 (76.2) | 165 (54.8) |
| **Currently live with a spouse or romantic partner** |  |  |
| Yes | 237 (14.2) | 8 (3.6)^b^ |
| No | 1767 (70.5) | 232 (77.1) |
| **Anyone who lives with you now who uses tobacco** |  |  |
| Cigarettes, cigars, cigarillos or filtered cigars and pipe tobacco | 391 (16.7) | 70 (22.2) |
| E-products exclusively | 35 (1.5) | 7 (2.1)^b^ |
| Other tobacco products, including smokeless, snus and hookah | 63 (2.3) | 15 (4.2) |
| No one living in the home uses tobacco | 1833 (74.2) | 207 (67.9) |
| **Last time you did the following two or more times: Lied or conned to get things or to avoid having to do something** |  |  |
| Past month | 362 (14.2) | 64 (19.9) |
| 2 to 12 months ago | 411 (16.4) | 75 (24.4) |
| Over a year ago | 391 (15.4) | 53 (16.3) |
| Never | 1272 (54.0) | 117 (39.4) |
| **Used alcohol in the past 12 months** |  |  |
| Yes | 1001 (46.6) | 177 (61.6) |
| No | 1433 (53.3) | 132 (38.4) |
| **Last time you had significant sleep trouble such as bad dreams, sleeping restlessly or falling asleep during the day** |  |  |
| Past month | 643 (26.2) | 100 (31.8) |
| 2 to 12 months ago | 413 (16.2) | 66 (20.9) |
| Over a year ago | 234 (9.5) | 30 (8.6) |
| Never | 1143 (47.9) | 112 (38.4) |
| **Race** |  |  |
| White alone | 1594 (67.4) | 219 (72.5) |
| Black alone | 479 (16.4) | 46 (12.7) |
| Other | 364 (16.2) | 44 (14.8) |
| **The views of people important to you on using ENDS** |  |  |
| Very positive | 53 (2.3) | 8 (1.6)^b^ |
| Positive | 126 (5.4) | 18 (6.1) |
| Neither positive nor negative | 468 (19.5) | 78 (23.3) |
| Negative | 723 (30.9) | 100 (35.6) |
| Very negative | 1054 (41.5) | 97 (31.1) |
| **Harmfulness of cigarettes to health** |  |  |
| Not at all harmful | 15 (0.6) | 5 (1.2)^b^ |
| Slightly harmful | 9 (0.7)^b^ | 2 (0.5)^b^ |
| Somewhat harmful | 68 (2.3) | 17 (7.6)^b^ |
| Very harmful | 615 (24.9) | 93 (27.8) |
| Extremely harmful | 1728 (71.5) | 192 (62.9) |
| **In past 30 days, noticed ENDS being advertised at gas stations, convenience stores, or other retail stores** |  |  |
| Yes | 1043 (44.8) | 169 (53.0) |
| No | 1386 (54.9) | 139 (46.7) |
| **Harmfulness of hookah to health** |  |  |
| Not at all harmful | 17 (0.7) | 6 (1.2)^b^ |
| Slightly harmful | 125 (5.8) | 19 (5.9) |
| Somewhat harmful | 517 (21.7) | 84 (30.2) |
| Very harmful | 740 (30.4) | 107 (33.9) |
| Extremely harmful | 1034 (41.2) | 93 (28.7) |
| **In past 30 days, noticed cigarettes or other tobacco products being advertised at fairs, festivals, or sporting events** |  |  |
| Yes | 76 (3.3) | 21 (6.3) |
| No | 2351 (96.1) | 284 (91.8) |
| **Gender** |  |  |
| Male | 1080(45.6) | 158(52.0) |
| Female | 1357 (54.4) | 151 (48.0) |
| **Harmfulness of ENDS compared to smoking cigarettes** |  |  |
| Less harmful | 559 (25.0) | 96 (32.6) |
| About the same | 1607(64.0) | 191 (61.6) |
| More harmful | 254 (10.1) | 21 (5.6) |
| **Currently enrolled in a degree program** |  |  |
| Yes | 1173 (49.4) | 171 (59.2) |
| No | 1257 (50.4) | 136 (40.2) |
| **Use of Anti-inflammatory or pain medication in the past 12 months^c^** |  |  |
| Yes | 1566 (64.8) | 228 (74.8) |
| No | 871 (35.2) | 81(25.2) |
| **Cigars are harmful to health^d^** |  |  |
| Yes | 2207 (90.2) | 265 (84.6) |
| No | 230(9.8) | 44 (15.4) |
| **In past 12 months, seen ENDS that claim to be less harmful** |  |  |
| Yes | 907 (39.2) | 140 (47.8) |
| No | 1525 (60.7) | 168 (51.8) |
| **Hookah susceptibility** |  |  |
| Yes | 622 (26.4) | 131 (41.8) |
| No | 1815 (73.6) | 178 (58.2) |

^a^Data is expressed as unweighted number (weighted percentage) of respondents.

^b^Relative standard error (RSE) >30%, therefore weighted percentages may not be reliable.

^c^The survey question was about taking anti-inflammatory or pain medication “In the past 12 months, did you take any of the following medications, even once?” Survey choices were Aspirin, Tylenol, Cox-2 inhibitors and other anti-inflammatory medications. Respondents selected using either Aspirin or Tylenol.

^d^Participants who responded that traditional cigars, cigarillos or filtered cigars were very harmful or extremely harmful to health were considered as perceiving that cigars were harmful to health.
