# Supplementary material for: Prospective predictors of electronic nicotine delivery system initiation in tobacco naive young adults: A machine learning approach
Source: Prev Med Rep. 2023 Feb 13;32:102148. doi: 10.1016/j.pmedr.2023.102148 (PMC9971268; doi:10.1016/j.pmedr.2023.102148)
Supplement: Supplementary data 5 [file mmc5.docx]

**Supplementary Table 2:** Association between ENDS initiation in Wave 5 and the significant baseline predictors (Wave 4) selected with ML.

|  | **Odds ratio^a^** |
| --- | --- |
| **ENDS susceptibility** |  |
| Yes | **3.02 (2.20 - 4.14)** |
| No | Ref |
| **How many days of physical activities specifically designed to strengthen your muscles^b^** | **1.16 (1.09 - 1.23)** |
| **How often visit social media accounts^c^** | **0.80 (0.72 - 0.89)** |
| **Used marijuana in the past 12 months** |  |
| Yes | **3.67 (2.61 - 5.17)** |
| No | Ref |
| **Cigarette susceptibility** |  |
| Yes | **2.63 (1.95 - 3.56)** |
| No | Ref |
| **Currently live with a spouse or romantic partner** |  |
| Yes | **0.23 (0.09 - 0.59)** |
| No | Ref |
| **Anyone who lives with you now who uses tobacco** |  |
| Cigarettes, cigars, cigarillos or filtered cigars and pipe tobacco | Ref |
| E-products exclusively | 0.98 (0.30 - 3.25) |
| Other tobacco products, including smokeless, snus and hookah | 1.43 (0.72 - 2.83) |
| No one living in the home uses tobacco | **0.68 (0.49 - 0.94)** |
| **Last time you did the following two or more times: Lied or conned to get things or to avoid having to do something** |  |
| Past month | Ref |
| 2 to 12 months ago | 1.05 (0.64 - 1.72) |
| Over a year ago | 0.74 (0.47 - 1.17) |
| Never | **0.53 (0.37 - 0.74)** |
| **Used alcohol in the past 12 months** |  |
| Yes | **1.88 (1.39 - 2.55)** |
| No | Ref |
| **Last time you had significant sleep trouble such as bad dreams, sleeping restlessly or falling asleep during the day** |  |
| Past month | Ref |
| 2 to 12 months ago | 1.07 (0.70 - 1.62) |
| Over a year ago | 0.76 (0.46 - 1.27) |
| Never | **0.65 (0.46 - 0.91)** |
| **Race** |  |
| White alone | Ref |
| Black alone | 0.73 (0.49 - 1.09) |
| Other | 0.84 (0.53 - 1.32) |
| **The views of people important to you on using ENDS** |  |
| Very positive | 0.94 (0.32 - 2.73) |
| Positive | 1.55 (0.87 - 2.76) |
| Neither positive nor negative | **1.57 (1.04 - 2.39)** |
| Negative | **1.54 (1.02 - 2.31)** |
| Very negative | Ref |
| **Harmfulness of cigarettes to health** |  |
| Not at all harmful | 2.62 (0.86 - 8.02) |
| Slightly harmful | 0.73 (0.08 - 6.72) |
| Somewhat harmful | **3.71 (1.65 - 8.35)** |
| Very harmful | 1.25 (0.92 - 1.70) |
| Extremely harmful | Ref |
| **In past 30 days, noticed ENDS being advertised at gas stations, convenience stores, or other retail stores** |  |
| Yes | **1.38 (1.08 - 1.75)** |
| No | Ref |
| **Harmfulness of hookah to health** |  |
| Not at all harmful | **2.85 (1.22 – 6.64)** |
| Slightly harmful | 1.46 (0.82 - 2.61) |
| Somewhat harmful | **2.01 (1.33 – 3.02)** |
| Very harmful | **1.60 (1.15 - 2.22)** |
| Extremely harmful | Ref |
| **In past 30 days, noticed cigarettes or other tobacco products being advertised at fairs, festivals, or sporting events** |  |
| Yes | **1.94 (1.03 - 3.65)** |
| No | Ref |
| **Gender** |  |
| Male | 1.29 (0.96 - 1.74) |
| Female | Ref |
| **Harmfulness of ENDS compared to smoking cigarettes** |  |
| Less harmful | Ref |
| About the same | 0.75 (0.55 - 1.02) |
| More harmful | **0.44 (0.24 - 0.80)** |
| **Currently enrolled in a degree program** |  |
| Yes | **1.53 (1.16 - 2.04)** |
| No | Ref |
| **Use of Anti-inflammatory or pain medication in the past 12 months** |  |
| Yes | **1.72 (1.28 - 2.32)** |
| No | Ref |
| **Cigars are harmful to health** |  |
| Yes | **0.61 (0.38 - 1.00)** |
| No | Ref |
| **In past 12 months, seen ENDS that claim to be less harmful** |  |
| Yes | **1.42 (1.04 - 1.94)** |
| No | Ref |
| **Hookah susceptibility** |  |
| Yes | **2.04 (1.53 - 2.72)** |
| No | Ref |
| **Hispanic** |  |
| Yes | 1.09 (0.79 - 1.53) |
| No | Ref |

Boldface indicates statistical significance

^a^Adjusted for gender, race and ethnicity and incorporated PATH complex design and weights except for the variables: race, gender and ethnicity, which are unadjusted odds ratios.

^b^Converted to continuous variables for the multivariable logistic regression models.

^c^Converted to continuous variables for the multivariable logistic regression models. Variable shows decreasing frequency of use from “More than once a day” to “Never”.
